# Adult Age Differences in Coping Strategies After the 2016 Flood

**DOI:** 10.1093/geroni/igaa057.1412

**Published:** 2020-12-16

**Authors:** Katie E Cherry, Matthew Calamia, Emily M Elliott, Quyen P Nguyen

**Affiliations:** Louisiana State University, Baton Rouge, Louisiana, United States

## Abstract

The 2005 Hurricanes Katrina and Rita had a devastating impact on south Louisiana, as did the more recent flooding in 2016. Multiple disaster exposures are associated with distress which may be lessened through adaptive coping behaviors, although prior disaster losses may affect current coping responses. In this study, we assessed self-reported coping strategies, resilience, and mental health outcomes after the 2016 flood. The sample was comprised of mostly middle-aged and older adults (N = 223, age range: 18-89 years). Three groups were compared: (1) non-flooded adults as controls, (2) once-flooded adults with structural damage to homes and property in 2016, and (3) twice-flooded adults who had relocated to Baton Rouge because of catastrophic losses in Hurricanes Katrina and Rita and they experienced damage in the 2016 flood. Analyses of variance confirmed that the three groups differed in coping responses, with non-flooded controls using significantly fewer strategies than their once and twice flooded counterparts. Correlation analyses demonstrated that age was positively associated with adaptive forms of coping (acceptance, religiosity) and negatively correlated with maladaptive coping (self-blame). These data suggest that awareness of prior severe weather experiences and catastrophic losses, which are likely for older adults living in hurricane prone areas, are an important consideration for disaster planning and the development of age-sensitive interventions to mitigate adversity.

